# Global Health on the Brink: The United States Withdrawal from WHO, Paris Climate Accord, and the 90-Day Freeze on Foreign Assistance: Implications and Strategies for Action

**DOI:** 10.24248/eahrj.v8i3.794

**Published:** 2025-01-30

**Authors:** Steve Wandiga, Léonard Ntakarutimana, Fabian Mashauri

**Affiliations:** a Kenya Medical Research Institute; b East African Health Research Commission

## Abstract

The recent executive orders by President Donald Trump to withdraw the United States (U.S.) from the World Health Organization (WHO) and the Paris Climate Agreement, compounded by a 90-day freeze on U.S. Foreign Assistance, present significant challenges to global health efforts. These actions threaten to exacerbate existing health crises, undermine decades of global health and health security gains, and leave Africa and the world more vulnerable to infectious diseases and public health threats. These decisions will likely hinder future health initiatives and disrupt critical climate change mitigation efforts. This short communication examines the potential consequences of these shifts and proposes strategies to mitigate their risks.

## BACKGROUND

The U.S. has historically played a crucial role in funding and supporting global health and climate initiatives. The withdrawal from WHO, disengagement from the Paris Climate Agreement, and the suspension of foreign assistance disrupt ongoing efforts to combat infectious diseases, strengthen health systems, and mitigate climate change. These decisions not only hinder current programs but also risk setting back long-term global health goals.

### WITHDRAWAL FROM THE WORLD HEALTH ORGANIZATION (WHO)

#### Funding Reductions

The U.S. has been the largest financial contributor to the WHO, providing approximately 18% of its budget^1^. The cessation of this funding jeopardizes the continuity of critical health programs, including those targeting HIV/AIDS, tuberculosis, and malaria, especially in low-income countries. Furthermore, the 90-day freeze on U.S. foreign assistance further strains existing programs, delaying essential interventions and threatening the delivery of life-saving treatments.

#### Pandemic Preparedness and Response

The U.S. withdrawal from WHO undermines global efforts to monitor and respond to health emergencies. The recall of U.S. personnel collaborating with WHO on health systems strengthening, health research, and disease surveillance, control, and response will impede the timely detection of and response to future pandemics, increasing the risk of widespread health crises.

#### Leadership Void

The U.S. exit creates a leadership vacuum, which may be filled by developed nations and other emerging countries and philanthropies to influence the direction of global health priorities and strategies in the WHO therefore contributing to the definition of new global health balances. This shift in influence could alter health policy decisions, potentially diverting resources away from pressing global health concerns.

#### WHO Governance Rethinking

The WHO was repetitively accused of mismanagement of the global health crisis including the COVID–19 pandemic, a too high political and economic dependency on member states and pharmaceuticals in decision-making, and it is fair to adopt reforms. The U.S. withdrawal could be a window of opportunity for WHO member states to rethink its legal and regulatory framework regarding the funding mechanisms and decision-making processes.

### WITHDRAWAL FROM THE PARIS CLIMATE AGREEMENT

#### Acceleration of Climate Change

The U.S. is the world’s second-largest emitter of greenhouse gases after China, and its withdrawal from the Paris Climate Agreement undermines international efforts to combat climate change. This disengagement could lead to the increased frequency and severity of climate-related health issues, such as heatwaves, flooding, respiratory diseases, and the spread of vector-borne diseases like malaria and dengue.

#### Economic and Health Impacts

Disengagement from the Paris Agreement may slow the U.S.’s transition to clean energy, perpetuating reliance on fossil fuels. This will likely worsen air quality, leading to respiratory and cardiovascular diseases, and escalating healthcare costs. The 90-day freeze on foreign assistance will exacerbate these health burdens, particularly in countries reliant on U.S. financial aid and technical support.

#### Global Climate Leadership Vacuum

The U.S. withdrawal weakens global climate initiatives, potentially leading other nations to scale back their commitments. This collective backslide could exacerbate global warming, with widespread health consequences, including among others, heat-related illnesses and deaths, increased food insecurity and displacement due to extreme weather events and high risk of emergence of new epidemics and pandemics; patterns shifting of infectious disease transmission, and worsening of the maternal and child health outcomes.

### IMMEDIATE AND LONG-TERM IMPLICATIONS OF THE 90-DAY FREEZE ON U.S. FOREIGN ASSISTANCE

#### Disruption of Health Programs

A significant portion of health programs across Africa, Asia, and Latin America depend on U.S. Foreign Assistance. These programs, which include efforts to combat infectious diseases including HIV/AIDS, tuberculosis, and malaria, will experience delays in resource distribution, hindering service delivery.

#### Emergency Health Response Impacts

Global health emergencies, such as disease outbreaks and humanitarian crises, rely on rapid U.S. aid. The freeze limits the ability of international organizations and governments to deploy resources and personnel swiftly, hampering response efforts to emerging health threats.

#### Strain on Health Infrastructure

U.S. aid supports local capacity-building programs, training healthcare professionals, and strengthening health systems. The freeze threatens these programs, placing further strain on already underfunded local health infrastructures.

#### Exacerbating Health Disparities

The freeze on U.S. Foreign Assistance disproportionately affects vulnerable populations, particularly in countries with high disease burdens and limited domestic funding. The disruption of essential health services will worsen health disparities, particularly for maternal and child health.

#### Strain on Global Health Initiatives

The U.S. plays a central role in funding global health initiatives such as the Global Fund and GAVI, the Vaccine Alliance. The freeze will disrupt these efforts, slowing progress toward achieving the U.N. Sustainable Development Goals related to health.

### STRATEGIES TO MITIGATE RISKS AND ENSURE CONTINUED PROGRESS

#### Advocate for Increased Domestic Investment

Encourage local governments to increase national health budgets, especially in regions like East Africa, to maintain and expand health services.

Promote public-private partnerships to drive local investments in health research and development.

#### Enhance Regional Collaboration

Form regional consortia in East Africa, partnering with organizations such as the African Academy of Sciences and Africa CDC, to pool resources and share expertise.

Build local partnerships with governments, academic institutions, and private sectors to co-invest in health programs and strengthen local health systems.

#### Diversify Funding Sources

Engage alternative international donors e.g., China, India, and the European Union (EU), along with global organizations like the Global Fund and Wellcome Trust.

Strengthen partnerships with pharmaceutical companies and philanthropies (e.g., the Gates Foundation) to secure funding for essential programs.

Leverage philanthropic support, including from organizations like the Clinton Health Access Initiative, for project-specific funding.

#### Leverage Innovative Funding Mechanisms

Use crowdfunding platforms to secure funding for specific research and health projects.

Pursue the commercialization of health innovations, such as diagnostic tools and vaccines, to generate revenue for reinvestment in local programs.

#### Focus on Capacity Building

Invest in training local researchers and healthcare professionals to reduce dependency on external expertise and build sustainable health systems.

Develop locally adapted technologies to address health needs in resource-limited settings.

#### Strengthen Data and Surveillance Systems

Utilize AI and machine learning for disease surveillance, predictive modeling, and early outbreak detection.

Create centralized data repositories to improve cross-border research and rapid responses to health crises.

#### Prioritize Multi-Disease and Climate-Health Research

Investigate the intersection of climate change and infectious diseases to attract funding for innovative research.

Adopt a multi-disease approach to address not only HIV/AIDS, TB, and malaria, but also emerging threats like antimicrobial resistance and zoonotic diseases.

#### Advocate for Policy Support

Use regional and global platforms (e.g., the African Union, EAC, WHO) to advocate for continued investment in global health.

Promote incentives for local businesses to invest in health research and innovation.

## CONCLUSION

The U.S. withdrawal from the WHO, the Paris Climate Agreement, and the 90-day freeze on foreign assistance pose significant challenges to global health and climate efforts. However, it also represents an opportunity for proactive and collaborative strategies that can help mitigate its negative impacts through exploring and implementing other alternatives, ensuring continued progress in addressing pressing health and climate issues worldwide. By addressing the immediate and long-term implications of these shifts, we can ensure continued progress in global health and climate initiatives, strengthening resilience for future generations.
